# Roles of Erythrocytes in Human Health and Disease 2.0

**DOI:** 10.3390/ijms25084446

**Published:** 2024-04-18

**Authors:** Francesco Misiti

**Affiliations:** Human, Social and Health Department, University of Cassino and Lazio Meridionale, V. S. Angelo, Loc. Folcara, 03043 Cassino, FR, Italy; f.misiti@unicas.it

This Special Issue titled “*Roles of Erythrocytes in Human Health and Disease 2.0*” delves into the intricate interplay between various factors, including COVID-19 infection, sickle cell disease, preeclampsia, reactive oxygen species, glucose control, favism, and tumor microenvironment, in red blood cell (RBC) biology. This special issue collected 11 papers (List of Contributors #1–11).

The main aim of this collection is to elucidate the underlying mechanisms and consequences of these influences on RBC function and physiology through a multidisciplinary approach encompassing cellular, biophysical, and biochemical analyses. Several studies show the direct involvement of erythrocytes in coronavirus infections [1,2]. Bosek et al. (#1) investigate the impact of COVID-19 on cellular factors governing RBC aggregation, employing dextran as a model system. By examining the potential causes and consequences of altered RBC aggregation dynamics in the context of COVID-19 infection, this study provides insights into the pathophysiology of the disease and its implications for hemorheology. Next, Chen et al. explore the biophysical profiling of sickle cell disease using an interesting approach utilizing deep learning and holographic cytometry (HC) (#2). By characterizing the morphological and mechanical properties of sickle RBCs, this research aims to enhance our understanding of disease progression and identify potential therapeutic targets. Sandor et al. (#3) investigate the influence of early-onset preeclampsia on perinatal RBC characteristics, shedding light on the prenatal factors shaping neonatal erythrocyte physiology and their implications for maternal and fetal health. Previous studies [3] highlight pathways involving oxidative stress and T2DM. The mechanochemical synergism of reactive oxygen species on the RBC membrane is examined by Kozlova et al. (#4), elucidating the intricate crosstalk between mechanical forces and biochemical pathways in modulating erythrocyte integrity and function. Additionally, the association between glucose control and circulating levels of RBC-derived vesicles in type 2 diabetes mellitus patients with atrial fibrillation is explored by Berezin et al. (#5). The structural and functional properties of favism erythrocytes are investigated by Dinarelli et al. (#6), uncovering specific metabolic adaptations and their implications for cellular aging and disease susceptibility. Bizjak et al. (#7) investigate alterations in hemorheological and hematological parameters in critically ill COVID-19 patients studied over a one-month observation period, elucidating the dynamic changes in blood rheology and composition in response to viral infection. Next, the adverse effects of cigarette smoke extract on RBCs via p38 MAPK-initiated, Fas-mediated eryptosis are elucidated in the article by Restivo et al. (#8), highlighting the detrimental impact of environmental toxins on erythrocyte homeostasis [4]. Omics markers are associated with RBC transfusion in trauma patients (LaCroix et al. #9), offering potential prognostic and therapeutic insights for managing traumatic injuries. Furthermore, the involvement of erythroid cells as active participants in the tumor microenvironment is discussed by Shevchenko et al. (#10), elucidating their role in tumor progression and immunomodulation. Lastly, the dual role of erythrocytes in health and disease via their adhesiveness is explored by Asaro et al. (#11), emphasizing the importance of understanding the context-dependent nature of erythrocyte interactions in physiological and pathological conditions. Through comprehensive investigation and integration of diverse methodologies, this Special Issue advances our understanding of the multifaceted influences on RBC biology and their implications for human health and disease management.
